# Effect of peripheral cellular senescence on brain aging and cognitive decline

**DOI:** 10.1111/acel.13817

**Published:** 2023-03-23

**Authors:** Vivekananda Budamagunta, Ashok Kumar, Asha Rani, Linda Bean, Sahana Manohar‐Sindhu, Yang Yang, Daohong Zhou, Thomas C. Foster

**Affiliations:** ^1^ Department of Neuroscience, McKnight Brain Institute University of Florida Gainesville Florida USA; ^2^ Genetics and Genomics Graduate Program, Genetics Institute University of Florida Gainesville Florida USA; ^3^ Department of Pharmacodynamics, College of Pharmacy University of Florida Gainesville Florida USA; ^4^ Department of Biochemistry and Structural Biology University of Texas Health Science Center at San Antonio San Antonio Texas USA

**Keywords:** aging, cognition, inflammation, oxidative stress, senolytic NMDA receptor, transcription

## Abstract

We examine similar and differential effects of two senolytic treatments, ABT‐263 and dasatinib + quercetin (D + Q), in preserving cognition, markers of peripheral senescence, and markers of brain aging thought to underlie cognitive decline. Male F344 rats were treated from 12 to 18 months of age with D + Q, ABT‐263, or vehicle, and were compared to young (6 months). Both senolytic treatments rescued memory, preserved the blood–brain barrier (BBB) integrity, and prevented the age‐related decline in hippocampal N‐methyl‐D‐aspartate receptor (NMDAR) function associated with impaired cognition. Senolytic treatments decreased senescence‐associated secretory phenotype (SASP) and inflammatory cytokines/chemokines in the plasma (IL‐1β, IP‐10, and RANTES), with some markers more responsive to D + Q (TNFα) or ABT‐263 (IFNγ, leptin, EGF). ABT‐263 was more effective in decreasing senescence genes in the spleen. Both senolytic treatments decreased the expression of immune response and oxidative stress genes and increased the expression of synaptic genes in the dentate gyrus (DG). However, D + Q influenced twice as many genes as ABT‐263. Relative to D + Q, the ABT‐263 group exhibited increased expression of DG genes linked to cell death and negative regulation of apoptosis and microglial cell activation. Furthermore, D + Q was more effective at decreasing morphological markers of microglial activation. The results indicate that preserved cognition was associated with the removal of peripheral senescent cells, decreasing systemic inflammation that normally drives neuroinflammation, BBB breakdown, and impaired synaptic function. Dissimilarities associated with brain transcription indicate divergence in central mechanisms, possibly due to differential access.

## Graphical abstract

The results of the current study demonstrate that senolytic treatment reduced morphological evidence of microglial activation, decreased expression of immune response genes in the dentate gyrus, and rescued hippocampal‐dependent spatial memory. Further, senolytic intervention preserved the blood‐brain barrier integrity. Finally, senolytic treatment prevented the age‐related decline in hippocampal N‐methyl‐D‐aspartate receptor‐mediated synaptic function associated with impaired cognition.

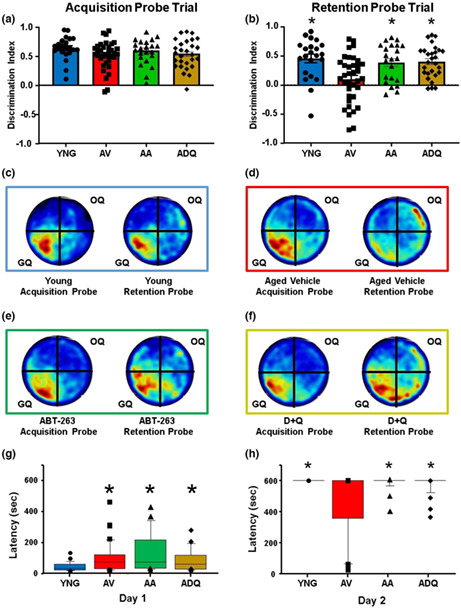

**Abbreviations**
AA: aged animals treated with ABT‐263
ADQ: aged animals treated with D+Q
ABT‐263: navitoclax
AGE‐RAGE: advanced glycation end products‐receptor for AGE
ANOVA: analysis of variance
AV: aged animals treated with vehicle
BBB: blood brain barrier
Bcl‐XL: B‐Cell lymphoma extra large
CA1: Cornu Ammonis 1
CA3: Cornu Ammonis 3
CNS: central nervous system
CXCL5: C‐X‐C motif chemokine 5
DAB 3: 3'‐diaminobenzidine
DAVID: database for annotation, visualization and integrated discovery
DEG: differentially expressed gene
D+Q: Dasatinib + Quercetin
DG: dentate gyrus
DI: discrimination index
DNA: deoxyribo nucleic acid
DNQX: 6,7‐dinitroquinoxaline‐2,3‐dione
EGF: endothelial growth factor
EPSP: excitatory post synaptic potential
ERCC: external RNA controls consortium
F344: Fisher 344
fEPSP: field excitatory post synaptic potential
FDR: false discovery rate
GABA: gamma aminobutyric acid
G‐CSF: granulocyte colony‐stimulating factor
GEO: gene expression omnibus
GO: gene ontology
GQ: goal quadrant
HIF‐1: hypoxia‐inducible factor‐1
IA: inhibitory avoidance
Iba‐1: allograft inflammatory factor 1
IFNγ: interferon gamma
IHC: immunohistochemistry
IL1‐α: interleukin 1 alpha
IL1‐β: interleukin 1 beta
IL‐2: interleukin 2
IL‐4: interleukin 4
IL‐5: interleukin 5
IL‐6: interleukin 6
IL‐12p70: interleukin 12p70
IL‐13: interleukin 13
IL‐17α: interleukin 17 alpha
IL‐18: interleukin 18
IP‐10: interferon gamma‐induced protein 10
KEGG: Kyoto Encyclopedia of Genes and Genomes
MAPK: mitogen‐activated protein kinase
mRNA: messenger ribo nucleic acid
MWM: Morris Water Maze
NF‐κB: nuclear factor kappa B
NMDA: N‐methyl‐D‐aspartate
NMDAR: N‐methyl‐D‐aspartate Receptor
OQ: opposite quadrant
PI3K/Akt: phosphatidylinositol 3‐kinase/protein kinase B
PROTAC: proteolysis targeting chimeric
PTX: picrotoxin
RANTES: chemokine ligand 5
Rap1: Ras‐Proximate‐1
RIN: RNA integrity number
RNA: Ribo nucleic acid
SASP: senescence associated secretory phenotype
SEM: standard error of mean
TBST: Tris‐buffered saline with 0.1% Tween
TGFβ: transforming growth factor beta
TNF: tumor necrosis factor
TNFα: tumor necrosis factor alpha
YNG: young

## 1 INTRODUCTION

Cellular senescence is a hallmark of aging and plays a crucial role in aging and a wide variety of age‐related diseases and disorders (Baker et al., 2011; Kirkland & Tchkonia, 2020). The senescence‐associated secretory phenotype (SASP) is an umbrella term that covers a wide variety of secreted proteins including inflammatory cytokines, growth factors, immune modulators, and tissue remodeling proteins (Coppe et al., 2010; Hernandez‐Segura et al., 2018). Considerable research has focused on paracrine influences of senescent cells, spreading senescence to neighboring cells, sometimes referred to as the bystander effect (da Silva et al., 2019; Nelson et al., 2018). However, a recent study demonstrated that senolytic treatment in humans reduced SASP factors in the plasma, suggesting SASP factors gain access to the circulation where they could have intra‐organ effects (Hickson et al., 2019).

The understanding that SASP factors enter blood circulation opens up a new avenue of research pertaining to the role of cellular senescence in various pathological conditions. One such interesting aspect to explore would be the impact of off‐site cellular senescence on cognitive function. Cognitive impairment, especially memory, has been studied widely with respect to cellular senescence, primarily in relation to senescence in the brain (Baker & Petersen, 2018; Lin et al., 2021; Sikora et al., 2021; von Zglinicki et al., 2021). Peripheral cellular senescence may contribute to chronic low‐grade systemic inflammation during aging (Budamagunta, Foster, & Zhou, 2021) and the exaggerated release of a variety of inflammatory mediators in response to inflammatory stimuli (Budamagunta, Manohar‐Sindhu, et al., 2021). In turn, systemic inflammation can drive brain changes that mimic aging (Barter et al., 2020, 2021; Mavrikaki et al., 2021), possibly contributing to the trajectory of cognitive decline (Barter et al., 2021; Bettcher & Kramer, 2014; Beydoun et al., 2019; Manabe & Heneka, 2022; Tampubolon, 2016; Zhao et al., 2019). Thus, cellular senescence accumulation in the peripheral tissues, in both age‐related and pathological contexts, could lead to impaired memory function.

We hypothesized that peripheral cellular senescence contributes to brain aging and age‐related cognitive impairment. Therefore, we compared the effects of dasatinib + quercetin (D + Q), which can cross the blood‐brain barrier (BBB; Guntner et al., 2020; Ishisaka et al., 2011; Porkka et al., 2008), decreasing oxidative stress, and clearing senescent cells in the brain (Ogrodnik et al., 2021; Zhang et al., 2019), and ABT‐263, which does not normally cross the BBB and has senolytic activity limited to peripheral tissues (Mehdipour et al., 2021; Yamaguchi & Perkins, 2012). The results indicate that treatment with ABT‐263 or D + Q decreased markers of peripheral senescence, including plasma SASP factors such as pro‐inflammatory cytokine levels. Senolytic treatments reduced morphological evidence of microglial activation, decreased expression of immune response genes in the dentate gyrus (DG), and rescued spatial memory and hippocampal synaptic transmission. ABT‐263 and D + Q maintained the integrity of the BBB; therefore, it is unlikely that clinically significant amounts of ABT‐263 crossed into the brain. Finally, differences in DG gene expression for TNF signaling, cell death, negative regulation of apoptosis, and microglial activation were consistent with differences in access of D + Q and ABT‐263 to the brain. The results indicate that peripheral senescence, through the elevation of plasma SASP factors, is an important contributor to several markers of brain aging and cognitive decline.

## 2 RESULTS

### 2.1 Behavior and cognitive function

Middle‐aged rats (12 months) were randomly divided into three groups that received a vehicle treatment (AV, *n* = 36), a dasatinib (1.2 mg/kg) + quercetin (12 mg/kg) cocktail treatment (ADQ, *n* = 28), or ABT‐263 (12 mg/kg) treatment (AA, *n* = 22). Young animals (6 months; YNG; *n* = 22) were tested at the same time as older groups for cue discrimination, episodic spatial memory on watermaze, and inhibitory avoidance. Aging is associated with impaired retention of hippocampal‐dependent memory for rapidly acquired spatial/contextual information, examined using the episodic memory version of the spatial watermaze task and inhibitory avoidance (Foster, 2012; Foster & Kumar, 2007; Guidi, Rani, et al., 2015; Kumar & Foster, 2013; Markowska et al., 1989; Martinez Jr. & Rigter, 1983; McGuiness et al., 2017; Vasquez et al., 1983).

#### 2.1.1 Cue discrimination training

**FIGURE 1:**
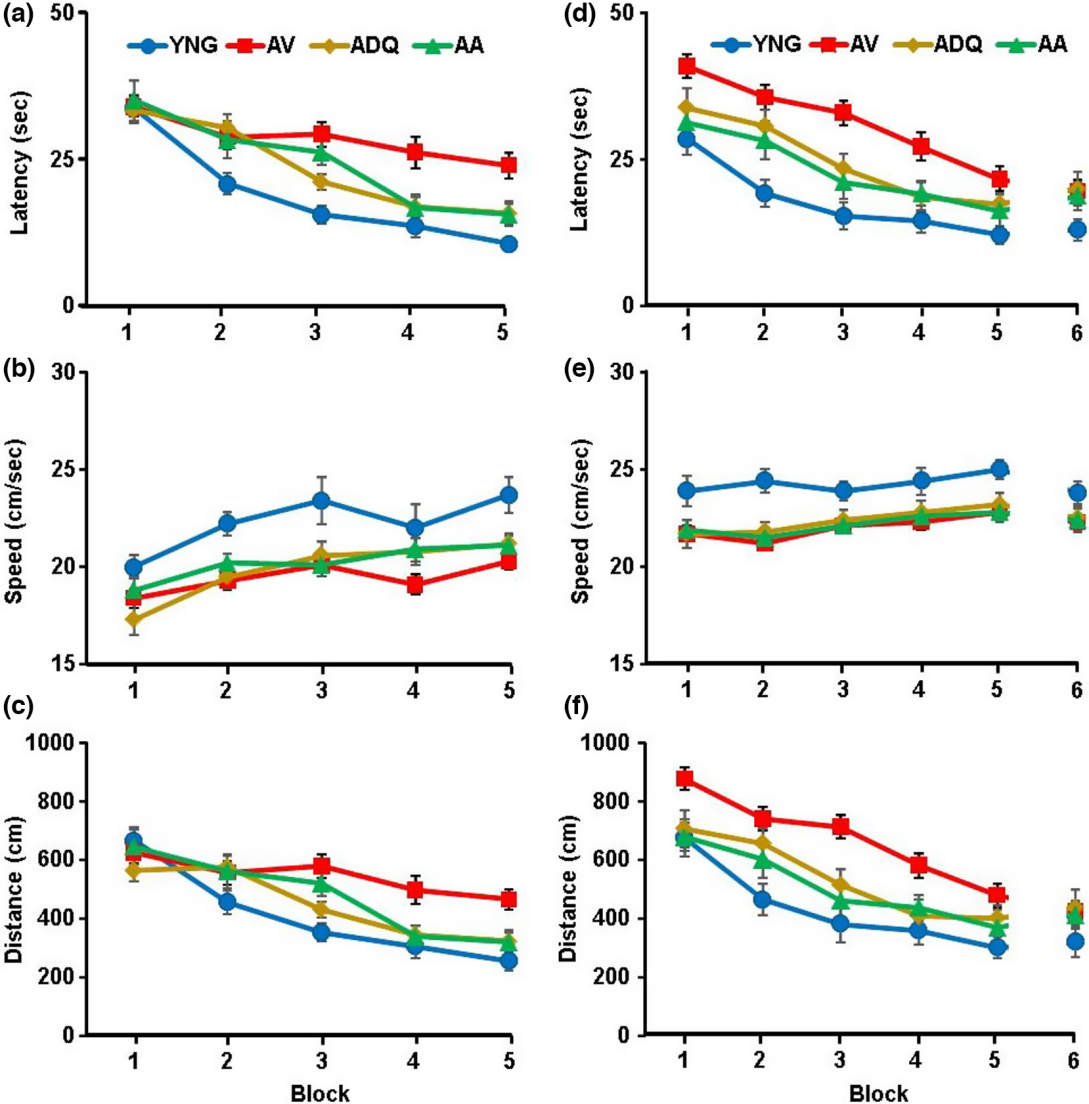
Senolytic treatments improve performance on the one‐day watermaze. (a,d) latency, (b,e) swim speed and (c,f) escape distance for each training block for the cue discrimination a‐c and spatial discrimination d and f tasks.

A repeated measures ANOVA of the latency to reach the platform across training trials for the cue discrimination task revealed a significant effect of training [*F*(4, 416) = 52.95; *p* < 0.0001], a group difference [*F*(3, 104) = 8.49; *p* < 0.0001], and interaction [*F*(12, 416) = 2.77; *p* < 0.005] (Figure 1a). Post hoc analyses indicated that the AV group had an increased latency relative to YNG, ADQ, and AA groups. YNG exhibited the shortest latency relative to all older age groups. The AA and ADQ groups were intermediate and not different from each other. Latency differences were due in part to a slower swim speed (Figure 1b). Swim speed increased with training [*F*(4, 416) = 17.53; *p* < 0.0001] and exhibited a group difference [*F*(3, 104) = 7.23; *p* < 0.0005]. Post hoc comparisons indicated YNG had a greater swim speed relative to the other groups and older animals were not different from each other. A repeated measures ANOVA on the distance to reach the platform across training trials for the cue discrimination task revealed a significant effect of training [*F*(4, 416) = 40.59; *p* < 0.0001], a group difference [*F*(3, 104) = 5.46; *p* < 0.005], and interaction [*F*(12, 416) = 2.56; *p* < 0.005] (Figure 1c). Post hoc analyses indicated that the AV group had an increased distance relative to the YNG and ADQ groups. Thus, all groups exhibited a decreased latency and pathlength with training, indicating learning of the cue discrimination; although differences due to treatment were noted with the shortest latency and pathlength for YNG and the longest latency and pathlength for AV animals.

#### 2.1.2 Spatial discrimination training

Three days following cue discrimination training, animals were tested on the one‐day version of the spatial watermaze, which is sensitive to age‐related impairment of episodic spatial memory (Foster, 2012). A repeated measures ANOVA on the latency of rats to reach the platform yielded a significant effect of training [*F*(5, 520) = 38.11; *p* < 0.0001] and treatment [*F*(3, 104) = 13.17; *p* < 0.0001] in the absence of an interaction. Similar to the cue task, post hoc analyses indicated that the AV group had an increased latency relative to YNG and the ADQ and AA groups. YNG exhibited a shorter latency relative to all older age groups. The AA and ADQ groups were intermediate and not different from each other (Figure 1d). Again, the latency difference was due, in part, to a slower swim speed [*F*(3, 104) = 6.84; *p* < 0.0005] (Figure 1e). Post hoc comparisons confirmed that YNG had a greater swim speed relative to the other groups and older animals were not different from each other. A repeated measures ANOVA performed on the pathlength to escape (distance) also yielded similar results with the four groups showing a significant effect of training [*F*(4, 520) = 35.96; *p* < 0.0001] and a group difference [*F*(3, 104) = 11.54; *p* < 0.0001] in the absence of an interaction (Figure 1f). Post hoc analyses confirmed that AV exhibited the longest distance relative to YNG, ADQ, and AA. The distance for ADQ was longer than YNG, and the AA group was not different from YNG or ADQ.

**FIGURE 2:**
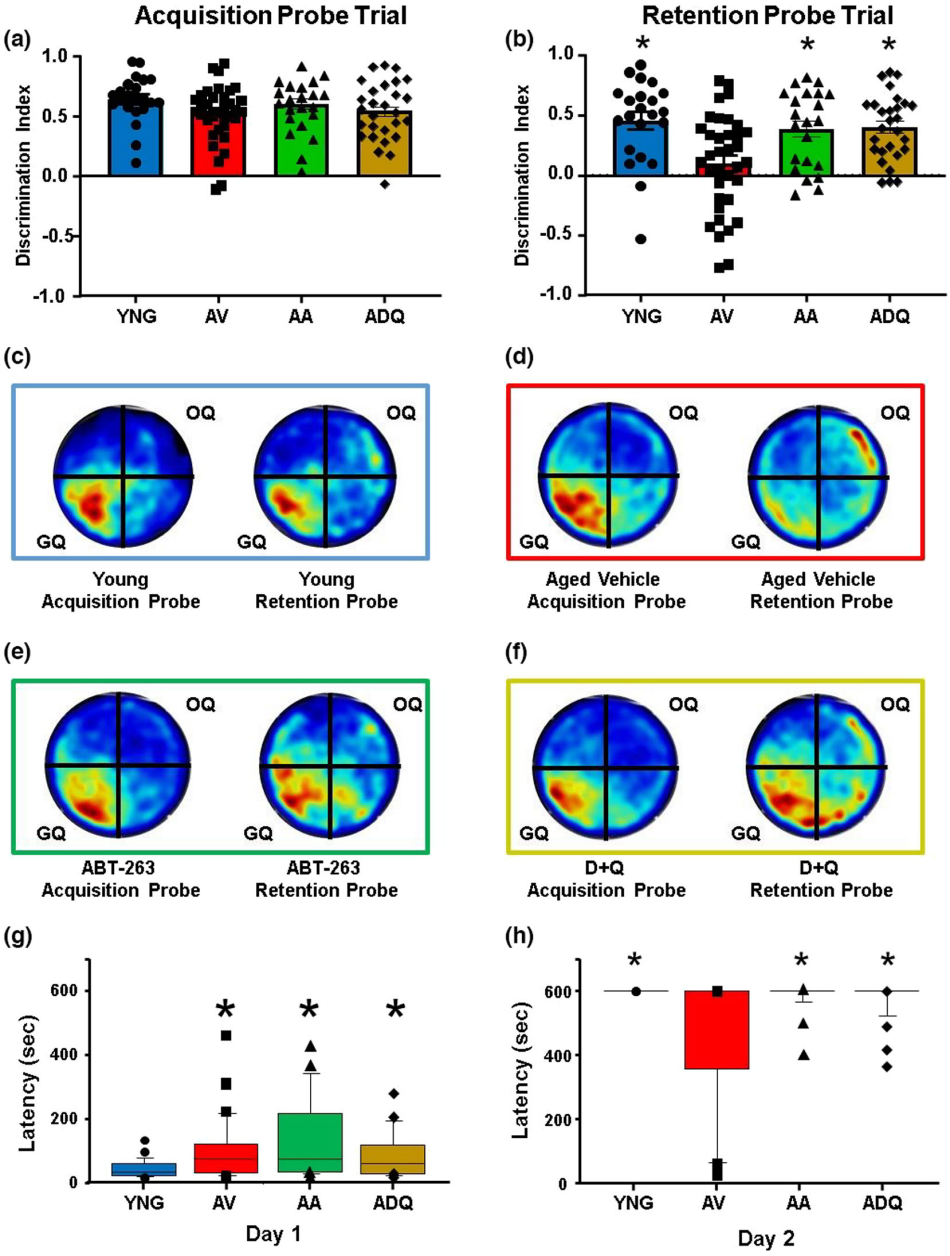
Senolytic treatments improve memory. Discrimination index (mean + SEM) for the (a) acquisition and (b) retention probe trials on the watermaze. (c–f) Group mean heat maps for the animals position during the acquisition and retention probe trials for (c) YNG, (d) AV, (e) AA, and (f) ADQ. Most animals directed their search to the goal quadrant (GQ). (g and h) Box plots for latency to enter the dark compartment of inhibitory avoidance during (g) Day 1 training and (h) Day 2 retention testing. The symbols indicate the mean (±SEM). Asterisks indicate a difference relative to AV (b, h) or YNG (g).

No group difference was observed for the acquisition probe trial discrimination index (DI) scores and one‐group *t* tests indicated that all groups performed above chance (*p* < 0.0001; Figure 2a). Examination of the DI scores for the 24‐hr retention probe trial indicated a significant group difference *F*(3, 104) = 6.77; *p* < 0.0005, and post hoc tests indicated that AV performed more poorly than all other groups (Figure 2b). Indeed, all groups, except AV exhibited a retention DI score well above chance (*p* < 0.0001), indicating impaired memory only for the AV group.

#### 2.1.3 Inhibitory avoidance

One week following spatial watermaze training, an inhibitory avoidance test was conducted. A Kruskal–Wallis one‐way ANOVA indicated a group difference (*H* = 8.20, *p* < 0.05) in the latency to cross into the dark chamber on the training day (Day 1). Post hoc test indicated an increased latency for all older groups relative to YNG (*p* < 0.05; Figure 2g). A Kruskal–Wallis test for latency to the dark chamber on the retention testing day was significant (*H* = 21.19, *p* < 0.0001), and post hoc tests indicated that the AV group had a shorter latency relative to the other three groups (Figure 2h), consistent with impaired memory. Interestingly, over 38% (14 animals) of the AV animals re‐entered the dark chamber at some point during the Day 2 retention testing trial, while none of the YNG entered in the allotted time and ~ 10% of ADQ (3 animals), and AA (2 animals) animals crossed into the dark chamber.

#### 2.1.4 Grip strength testing

Immediately before tissue collection, a subset of animals was weighed and tested for grip strength. An ANOVA indicated a difference in weight across groups [*F*(3, 88) = 8.63; *p* < 0.0001] due to increased weight of AV (*n* = 29, 440.45 ± 7.30 g) and AA (*n* = 22, 426.09 ± 5.18) relative to YNG (*n* = 22, 397.77 ± 6.62). In addition, ADQ (*n* = 19, 412.05 ± 5.07) animals weighed less than AV. The increase in body weight with age (~10%) is similar to previous reports for male F344 rats (Greenberg & Boozer, 2000; Turturro et al., 1999). Grip strength, normalized to weight, was different across groups [*F*(3, 88) = 238.18; *p* < 0.0001]. Post hoc test indicated that grip strength was increased in YNG, ADQ, and AA relative to AV and decreased in AA relative to YNG and ADQ (Figure S1).

### 2.2 Effect of senolytic treatment on markers of senescence in the periphery

#### 2.2.1 Gene expression

**FIGURE 3:**
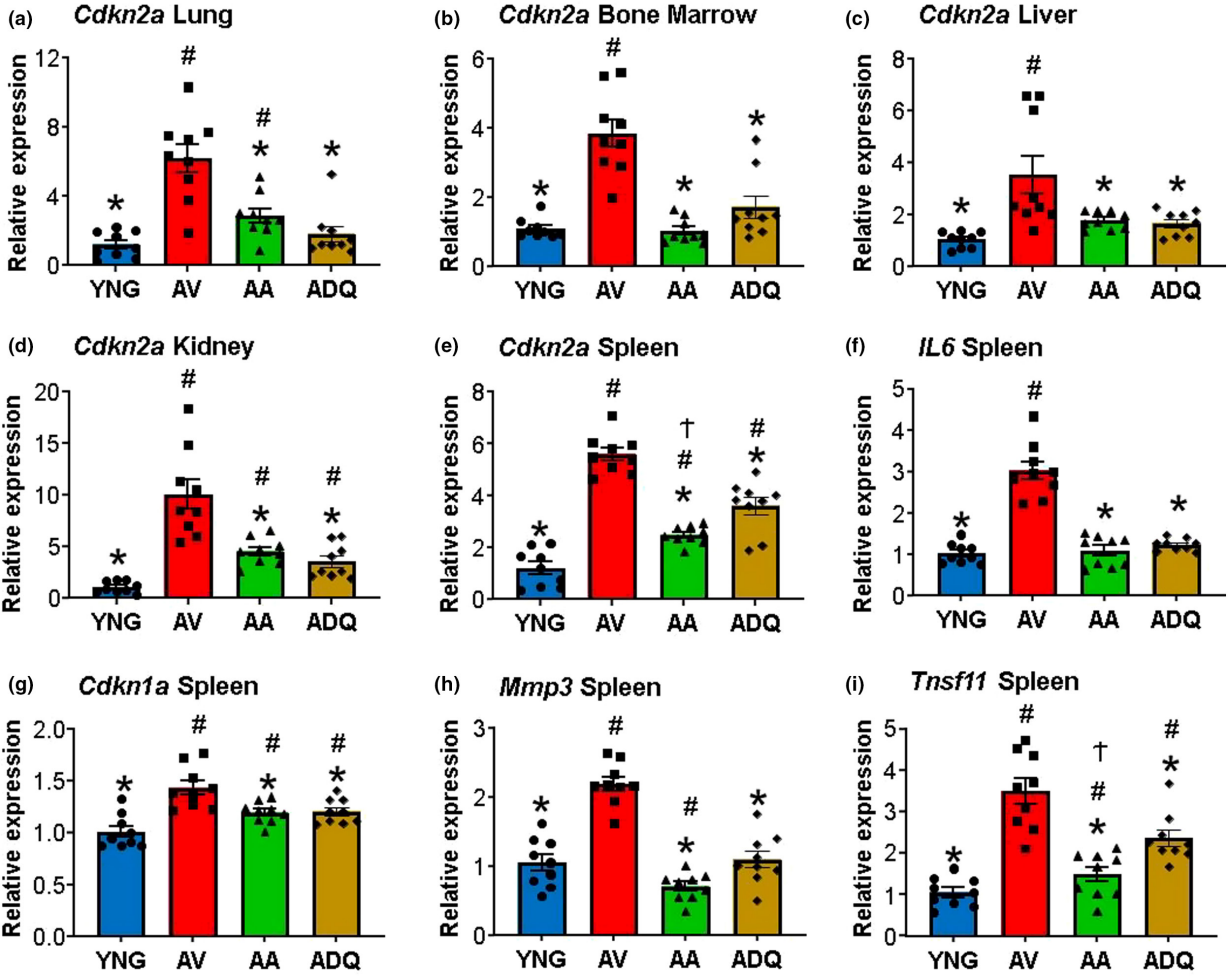
Senolytic treatment decreases expression of senescent and SASP genes in the periphery. Relative expression level of *Cdkn2a* in (a) lung, (b) bone marrow, (c) liver, (d) kidney, and (e) spleen. Relative expression level of (f) *IL* *‐6*, (g) *Cdkn1a*, (h) *Mmp3*, and (i) *Tnfsf11* in spleen. Error bars denote SEM (*n* = 9). Asterisks indicate a difference relative to AV, # denotes a difference relative to YNG, and Ϯ indicates a difference relative to ADQ (*p* < 0.05)

Nine animals in each group were used to examine qPCR expression of senescence and SASP genes as markers of senescent cell burden (He et al., 2020; Hernandez‐Segura et al., 2018; Sharpless & Sherr, 2015). ANOVAs indicated a treatment effect (*p* < 0.0005) for each measure. For the cyclin‐dependent kinase inhibitor 2A, *Cdkn2a* (P16^ink4a^), a group effect was observed in lung [*F*(3, 32) = 17.68, *p* < 0.0001], liver [*F*(3, 32) = 8.09, *p* < 0.0005], bone marrow [*F*(3, 32) = 24.85, *p* < 0.0001], kidney [*F*(3, 32) = 22.69, *p* < 0.0001], and spleen [*F*(3, 32) = 58.09, *p* < 0.0001] with the greatest expression in AV, and significantly decreased expression in YNG, AA, and ADQ (Figure 3a–e). *Cdkn2a* expression in senolytic‐treated groups was either not different from YNG (bone marrow and liver) or was intermediate between AV and YNG, such that expression in spleen and kidney was elevated relative to YNG. In addition, ABT‐263 had a greater effect in reducing expression of spleen *Cdkn2a*, relative to ADQ (Figure 3e). A similar pattern of aging and senolytic treatment was observed for other senescent and SASP‐related genes examined in the spleen (Figure 3f–i), such that for senolytic‐treated groups, *IL‐6* expression [*F*(3, 32) = 50.77, *p* < 0.0001] was decreased relative to AV and not different from YNG, and expression of *Cdkn1a* [*F*(3, 32) = 11.72, *p* < 0.0001] was intermediate and different from AV and YNG. Finally, ABT‐263 had a greater effect in reducing spleen expression of *Mmp3* [*F*(3, 32) = 38.79, *p* < 0.0001] and *Tnfsf11* [*F*(3, 32) = 25.95, *p* < 0.0001], relative to YNG and ADQ, respectively (Figure 3h,i).

#### 2.2.2 Plasma measures of cytokines and chemokines

Table 1 shows hormone, cytokine, and chemokine concentrations in the plasma of young and aged rats from the various treatment groups (*n* = 3–6 per group). A subset of pro‐inflammatory molecules (eotaxin, G‐CSF, IL‐1α, IL‐4, IL‐6, IL‐13, MCP‐1, and MIP‐2α) exhibited group differences (ANOVA *p* < 0.05), and post hoc tests indicated increased expression in all older groups relative to YNG. In contrast, IL‐12p70 was elevated in YNG relative to all older groups. Another group of pro‐inflammatory cytokines/chemokines (IL‐1β, IP‐10, RANTES) was increased in AV relative to YNG and senolytic‐treated groups, which were not different from each other. In a few cases, D + Q and ABT‐263 were differentially effective in reducing cytokines. IFNγ was increased in AV and ADQ relative to YNG, leptin was increased in all older groups and in ADQ relative AA, and a tendency (*p* = 0.067) for a group difference for EGF was due to increase expression in AV relative to YNG and AA groups. Finally, a group difference for TNFα was due to decreased expression in the ADQ group relative to AA and AV.

**TABLE 1:** Age and treatment effect on chemokines, cytokines, and hormone concentration.

Analyte	YNG	AV	ADQ	AA
Cxcl5	498.49 ± 105.16	377.13 ± 41.03	364.87 ± 31.69	468.00 ± 86.04
EGF	324.14 ± 74.77	746.68 ± 126.15^ac^	564.40 ± 97.29	420.26 ± 132.27
Eotaxin	5.67 ± 1.54	17.85 ± 1.95^a^	18.72 ± 2.49^a^	17.06 ± 0.94^a^
Fractalkine	77.80 ± 13.06	65.39 ± 8.82	55.80 ± 6.59	45.80 ± 5.20
G‐CSF	4.31 ± 1.57	41.12 ± 3.68^a^	44.57 ± 4.41^a^	46.98 ± 5.08^a^
IFNγ	162.66 ± 93.49	315.55 ± 33.45^a^	318.49 ± 10.91^a^	259.64 ± 22.23
IL‐1α	33.73 ± 8.26	153.90 ± 10.35^a^	150.45 ± 15.56^a^	152.72 ± 8.11^a^
IL‐1β	42.36 ± 9.09	148.91 ± 27.72^abc^	74.71 ± 8.17	58.75 ± 5.50
IL‐2	117.02 ± 12.79	113.37 ± ±15.52	89.37 ± 7.91	106.48 ± 12.78
IL‐5	79.78 ± 11.19	304.77 ± 91.20	98.76 ± 1.26	395.99 ± 195.79
IL‐4	23.06 ± 5.66	69.36 ± 7.02^a^	58.98 ± 3.75^a^	66.33 ± 7.57^a^
IL‐6	475.8 ± 113.35	2074.72 ± 285.94^a^	1734.62 ± 184.72^a^	2595.51 ± 737.2^a^
IL‐12p70	289.97 ± 34.54	156.23 ± 23.49^a^	130.62 ± 16.91^a^	151.23 ± 21.22^a^
IL‐13	11.93 ± 5.48	31.75 ± 4.17^a^	31.46 ± 4.62^a^	32.47 ± 3.86^a^
IL‐17α	33.00 ± 4.97	35.28 ± 5.34	47.74 ± 3.98^a^	44.15 ± 5.29
IL‐18	387.79 ± 28.84	351.78 ± 33.50	257.33 ± 24.16^a^	233.28 ± 16.60^a^
IP‐10	243.01 ± 29.83	479.34 ± 58.88^abc^	283.64 ± 16.18	274.97 ± 35.23
Leptin	12121.17 ± 4558.92	46226.94 ± 4210.80^a^	49742.82 ± 6776.02^ac^	32168.80 ± 6272.00^ab^
MCP‐1	594.18 ± 119.04	1057.22 ± 25.03^a^	923.31 ± 76.16^a^	937.37 ± 56.96^a^
IMP‐1 α	19.68 ± 3.85	28.22 ± 6.25^b^	16.95 ± 0.83	17.31 ± 0.09
MIP‐2	21.65 ± 3.84	89.86 ± 3.52^a^	88.75 ± 5.93^a^	86.72 ± 5.14^a^
RANTES	851.98 ± 219.69	9341.40 ± 3936.76^abc^	1321.07 ± 180.84	1322.47 ± 243.11
TNFα	5.27 ± 1.22	8.00 ± 0.67^b^	5.65 ± 0.78	8.62 ± 1.02^b^
VEGF	71.55 ± 25.53	138.11 ± 89.09	30.11 ± 10.85	152.15 ± 83.20

*Note*: ^a^ *p* <0.05 versus YNG, ^b^ *p* <0.05 versus ADQ, ^c^ *p* <0.05 versus AA. Data is represented as mean ± SEM (*n* = 3–6).

### 2.3 Senolytic treatment maintains synaptic function

**FIGURE 4:**
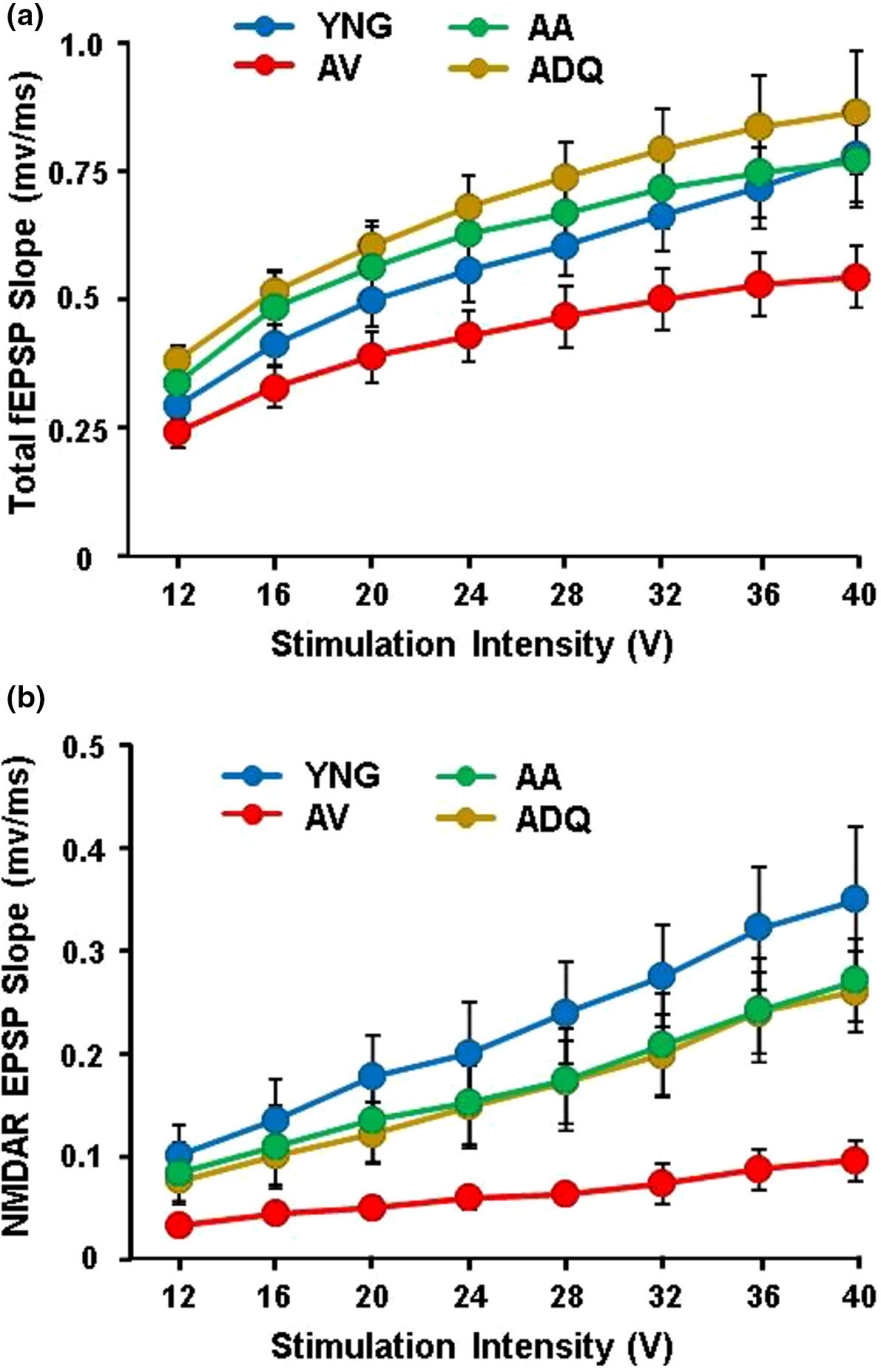
Senolytic treatment preserves synaptic function in the hippocampus. Slope of excitatory post synaptic field potentials recorded from hippocampal CA3‐CA1 synapses. The initial slope of the (a) total fEPSP and (b) NMDAR‐mediated component of the EPSP was measured for increasing stimulation intensity and input–output curves were generated. Each point represents the mean (±SEM) for the given stimulation intensity.

Age‐related impairment in episodic memory is linked to a decline in N‐methyl‐D‐aspartate receptor (NMDAR) function and associated changes in synaptic plasticity (Foster, 2012; Guidi, Rani, et al., 2015; Kumar et al., 2018; Kumar & Foster, 2013). Hippocampal CA1‐CA3 synaptic strength was examined by recording total‐ extracellular synaptic field potentials (fEPSPs) and generating input–output curves and plotting the slope of total synaptic response across the different stimulation intensities for YNG (*n* = 8/4 slices/animals), AV (*n* = 12/6 slices/animals), AA (*n* = 6/3 slices/animals), and ADQ (*n* = 7/4 slices/animals). A repeated measures ANOVA on the total CA3‐CA1 synaptic response to varying stimulation intensities indicated an effect of stimulation intensity [*F*(7, 203) = 86.22; *p* < 0.0001] and a group difference [*F*(3, 29) = 3.90; *p* < 0.05] in the absence of an interaction. Post hoc analyses revealed decreased synaptic strength in the AV group compared to ADQ and AA, with a tendency (*p* = 0.079) for a difference relative to YNG. No difference was observed in the total synaptic response between YNG and the two senolytic groups (Figure 4a).

Following collection of the total synaptic responses, the NMDAR‐mediated synaptic response was isolated. A repeated measures ANOVA on the NMDAR‐synaptic response indicated an effect of stimulation intensity [*F*(7, 203) = 101.91; *p* < 0.0001] and a group difference [*F*(3, 29) = 5.58; *p* < 0.005] and an interaction [*F*(21, 203) = 10.22; *p* < 0.0001], with the greatest differences observed toward the higher intensities of stimulation. Post hoc analyses revealed decreased NMDAR‐synaptic responses in the AV group compared to YNG, ADQ, and AA (Figure 4b). Again, no difference was observed between the YNG and either of the aged senolytic‐treated groups.

### 2.4 Similarities and differences in senolytic treatment on gene expression

**FIGURE 5:**
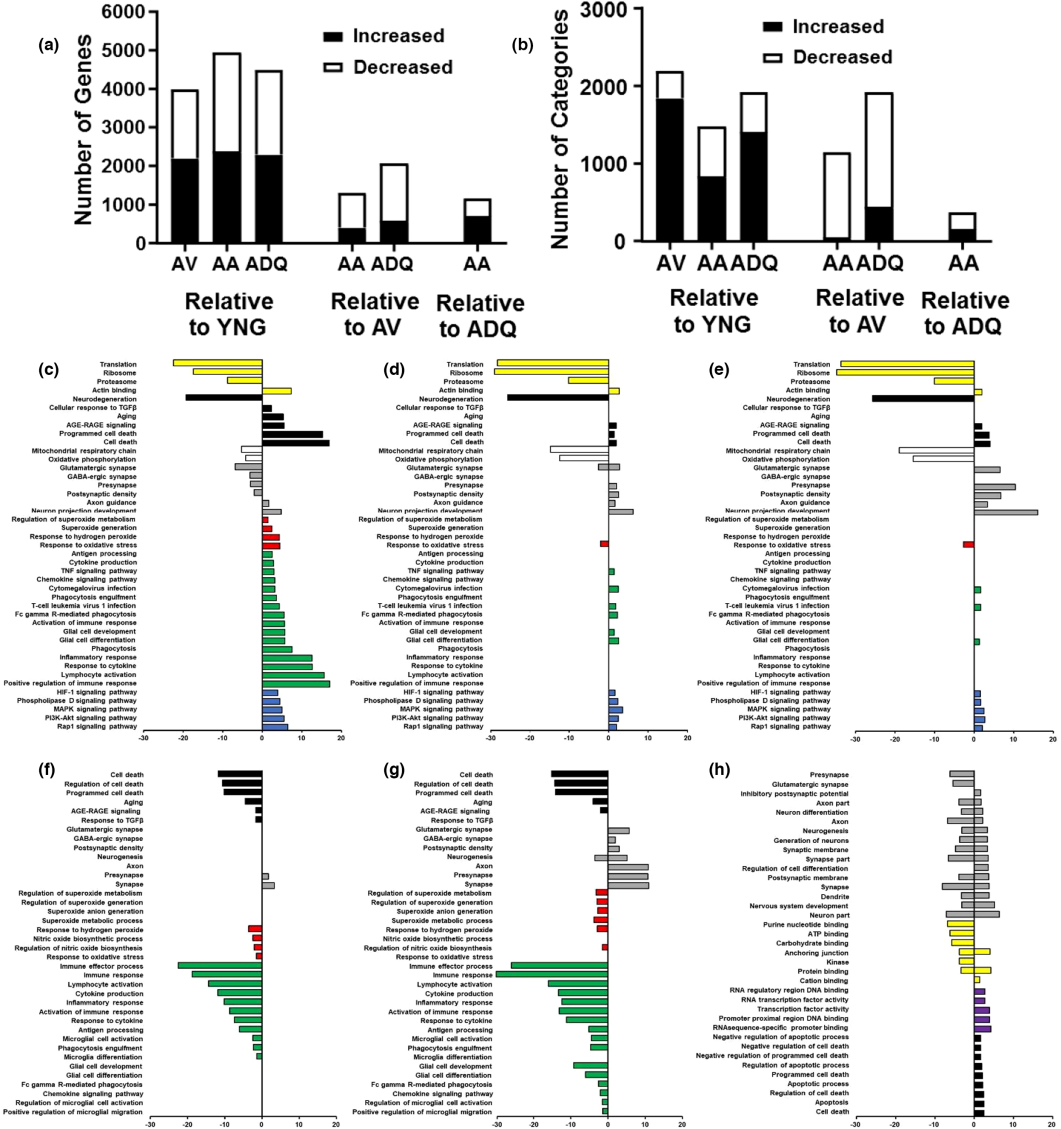
Effect of senolytic treatment on gene expression in the DG. (a) Graphic summary of the number of differentially expressed genes, which were either significantly increased (filled) or decreased (open) in relation to other groups. (b) Graphic summary for the number of GO categories identified for differentially expressed genes. (c–g) Bars represent the –log(adj‐*p* value) and log(adj‐*p* value) for selected GO term clusters for genes that increased or decreased compared to YNG for (c) AV, (d) AA, and (e) ADQ; and compared to AV for (f) AA and (g) ADQ, and (h) in AA compared to ADQ. GO terms were loosely grouped into categories: protein expression and binding (yellow), aging, age‐related disease, and cell death (black), mitochondria (open), neuronal/synaptic (gray), oxidative stress (red), glial and immune response (green), signaling pathways (blue), and RNA or DNA and molecular binding (purple).

A subset of animals was prepared for next‐generation sequencing of the DG (YNG, *n* = 10, AV, *n* = 12; AA, *n* = 11, ADQ, *n* = 11). Figure 5 illustrates the number of differentially expressed genes (DEGs) and number of enriched gene categories associated with the DEGs. All older groups expressed many more DEGs when contrasted with YNG, relative to differences in expression between other older groups (Figure 5a). In addition, senolytic treatments resulted in a marked shift in the number of upregulated and downregulated gene enrichment categories when comparing YNG and AV groups (Figure 5b). Compared to YNG, older groups, particularly AV, exhibited more categories for upregulated genes than for downregulated genes. In contrast, for DEGs between AV and senolytic‐treated groups, many more categories were observed for downregulated genes and the fewest number of gene enrichment categories was observed between senolytic treatment groups. Finally, compared to AV, D + Q influenced ~2 times more genes and categories than the ABT‐263 treatment.

For genes upregulated in each of the older groups relative to YNG (Figure 5c–e), functional annotation clustering analysis indicated that, across all age groups, increased expression was observed for actin binding, cell death and programed cell death, cytomegalovirus and T‐cell leukemia virus infection, antigen processing, glial cell differentiation, neuron projection development, axon guidance, and several signaling pathways; Rap1, MAPK, HIF‐1, PI3K‐Akt, phospholipase D, and AGE‐RAGE signaling pathway in diabetic complications (Figure 5c–e). Consistent with previous research, AV animals, but not senolytic‐treated animals, exhibited upregulation of genes associated with neuroinflammation (immune response, response to cytokine, inflammatory response, lymphocyte activation, and phagocytosis), including signaling pathways for regulation of cytokine production or chemokine signaling. Similarly, AV animals exhibited increased expression of genes linked to oxidative stress (response to oxidative stress, positive regulation of superoxide production, and superoxide metabolic process), and aging. Increased gene expression was observed for the transforming growth factor beta (TGFβ) receptor signaling pathway, consistent with an age‐related BBB dysfunction (Chen et al., 2020; Senatorov Jr et al., 2019).

In contrast, upregulated genes for ADQ relative to YNG did not include enrichment for most categories linked to immune activation, oxidative stress, aging, or TGFβ signaling (Figure 5e). Similarly, differential expression of genes linked to immune response, oxidative stress, aging, and TGFβ signaling was mainly absent from AA relative to YNG. However, similar to AV animals, AA animals exhibited increased expression of genes for TNF signaling, and Fc gamma R‐mediated phagocytosis, and glial cell development compared to YNG (Figure 5d).

Compared to YNG, all older groups exhibited decreased expression for genes linked to translation, ribosomes, proteasome, oxidative phosphorylation, and mitochondrial respiratory chain. In addition, all older groups exhibited decreased expression of pathways of neurodegeneration (Figure 5c–e). When comparing AV relative to YNG for downregulated genes, we observed enrichment of synaptic component genes including the glutamatergic, GABAergic and presynaptic and postsynaptic components (Figure 5c). In contrast, presynaptic and postsynaptic gene expression was increased in ADQ and AA groups relative to YNG. Genes for the glutamatergic synapse increased for ADQ and exhibited increased and decreased expression in the AA group (Figure 5d).

Comparison of gene expression across the older groups indicated that expression of genes for the category aging (GO:0007568) was decreased in AA and ADQ relative to AV (Figure 5f,g) and was not different across senolytic treatment groups (Figure 5h). Clusters linked to apoptosis and cell death were decreased in senolytic‐treated groups relative to AV; however, comparison of AA and ADQ groups indicated that expression of apoptosis and cell death genes, including those for the negative regulation of cell death and negative regulation of apoptosis, were elevated in the AA group relative to the ADQ group. Genes linked to oxidative stress were not different between AA and ADQ groups (Figure 5h) and ABT‐263 or D + Q treatment decreased genes linked to response to hydrogen peroxide, and regulation of nitric oxide biosynthesis relative to AV. However, D + Q may have had a greater effect in regulating oxidative stress linked to superoxide generation and metabolism, while ABT‐263 had more of an effect decreasing gene expression for nitric oxide biosynthetic processes (Figure 5f,g). Similarly, immune response clusters were not different between ADQ and AA groups (Figure 5h) and treatment with ABT‐263 or D + Q decreased genes linked to immune response relative to AV. However, D + Q treatment, but not ABT‐263 treatment, was associated with decreased expression of genes linked to positive regulation of glial cell migration and regulation of microglial cell activation (Figure 5f,g). Finally, examination of the top categories that were upregulated or downregulated or both up and downregulated in AA relative to ADQ, revealed that the AA group exhibited both up and downregulation of synaptic/neuronal genes and genes linked to molecular binding, and the AA group exhibited upregulation for cell death, apoptosis and negative regulation of cell death and apoptosis (Figure 5h).

### 2.5 Effect of senolytic treatment on morphological measures of microglial activation

**FIGURE 6:**
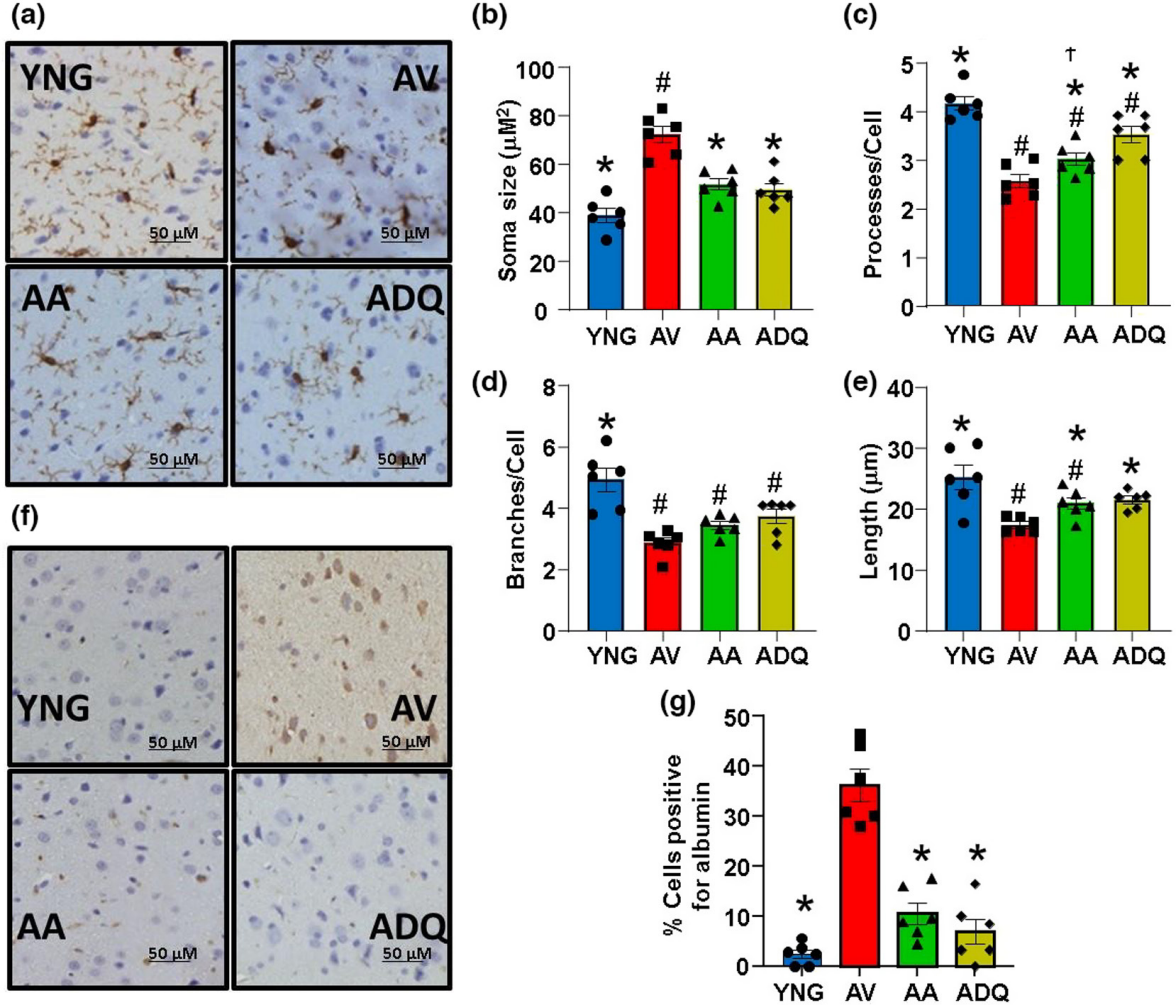
Senolytic treatment reduces age‐associated microglial activation. (a) Representative cortex sections immunohistologically stained for iba‐1. Mean ± SEM for microglial morphological parameters (b) soma size, (c) processes per cell, (d) branches per cell, and (e) the average length of the process. (f) Representative cortex sections immunohistologically stained for albumin. (g) Bars represent mean ± SEM. Asterisks indicate a difference relative to AV, # denotes a difference relative to YNG, and Ϯ indicates a difference relative to ADQ (*p* < 0.05).

Transcriptomic profiling indicated senolytic treatments decreased immune response genes. Differences were noted in that D + Q was better able to prevent the age‐related increase in genes for some inflammation signaling (i.e. TNF signaling and Fc gamma R‐mediated phagocytosis), glial cell development and migration, and regulation of microglial cell activation. To visualize microglia and morphologically examine activation status, we stained sections of cerebral cortex with Iba‐1 antibody (Figure 6a). The stained sections were subsequently analyzed for 25 cells/area of cerebral cortex across 6 areas (*n* = 150 cells cumulatively) measuring soma size, number of processes per cell, average length of these processes, and number of major branches per cell (Figure 6b–e). The measures were then averaged for each animal. Across most measures of microglial activation, greater activation was observed for AV (greater soma size, decreased number of processes, and reduced process length) and YNG exhibited the least microglia activation, with intermediate levels for senolytic‐treated animals. ANOVAs indicated a group difference for the size of the soma [*F*(3, 20) = 20.45; *p* < 0.0001], and post hoc tests indicated that all groups were different from the other groups except for the two senolytic groups, which were similar to each other. An ANOVA on the number of processes indicated a difference across groups [*F*(3, 20) = 23.73; *p* < 0.0001], and post hoc tests indicate that each group was different from the other three groups, with a decrease in the number of processes for AA animals relative to ADQ. For the number of branches [*F*(3, 20) = 12.38; *p* < 0.0001], post hoc tests indicated a decrease in number of branches of all older groups relative to YNG. Finally, for the length of processes [*F*(3, 20) = 8.29; *p* < 0.001], post hoc tests indicated that AV had decreased length relative to the other three groups and AA had a decrease in length relative to YNG.

### 2.6 Senolytic treatment maintains blood–brain barrier integrity

Albumin is peripherally restricted and enters the brain upon BBB disruption (Kassner & Merali, 2015). Cortical sections were stained for albumin (Figure 6f) to assess the percentage of cells positively stained for albumin for each animal (*n* = 6 per group). An ANOVA indicated a significant difference across groups [*F*(3, 20) = 39.43; *p* < 0.0001] and post hoc tests indicated a higher proportion of positively stained cells in the cerebral cortex of AV when compared to all the other groups (*p* < 0.0001; Figure 6g). The results for albumin staining and gene expression of TGFβ signaling indicate that senolytic treatment, from 12 to 18 months of age, can maintain the integrity of the BBB. Thus, leakage of clinically significant amounts of ABT‐263 into the brain is unlikely.

## 3 DISCUSSION

Long‐term treatment with ABT‐263 or D + Q, initiated in middle‐age, decreased peripheral markers of tissue senescence and systemic inflammation, and preserved hippocampal‐dependent cognitive function and NMDAR‐mediated synaptic transmission to a similar extent. This is the first demonstration that senolytic treatment preserves NMDAR function and provides novel information on the effect of ABT‐263 on plasma cytokines. Compared to older controls, senolytic treatments decreased transcription of DG genes linked to oxidative stress and immune response, and increased the expression of synaptic genes. However, D + Q had a greater effect on brain transcription categories associated with cellular senescence, decreasing expression of genes linked to apoptosis, regulation of apoptosis, and microglial activation that were not significant for ABT‐263 treatment. Dissimilarities associated with brain transcription indicate divergence in central mechanisms, possibly due to differential brain access. Previous work indicates that dasatinib enters the central nervous system (Guntner et al., 2020; Porkka et al., 2008) to clear senescent cells (Ogrodnik et al., 2021; Zhang et al., 2019). In contrast, ABT‐263 does not cross the BBB (Yamaguchi & Perkins, 2012), which may explain differential effects. In particular, senescent cells are resistant to apoptosis due to upregulation of anti‐apoptotic pathways. Relative to the ADQ group, the AA group exhibited increased expression of genes for apoptosis and negative regulation of apoptosis, consistent with the idea that senescent cells are primed to undergo apoptosis, while the implementation of the death program is provisionally suppressed (Fan et al., 2020).

Cellular senescence is also associated with increase oxidative stress (Doubleday et al., 2020; Passos et al., 2007; Rajapakse et al., 2011) and elimination of senescent cells by D + Q may contribute to the decreased expression of genes for superoxide metabolic pathways in the brain. Alternatively, quercetin also passes the BBB and has anti‐oxidant properties (Ishisaka et al., 2011), which may contribute to greater downregulation of superoxide metabolic pathways, specifically by D + Q.

Previous work suggests that a failure of large doses of ABT‐263 (50 mg/kg) to reduce established microglia activation in aged mice was due to the inability of ABT‐263 to cross the BBB (Mehdipour et al., 2021). In the current study, long‐term treatment with ABT‐263 or D + Q decreased the age‐related morphological markers of microglial activation and expression of immune response genes. In the case of ABT‐263, the effect may have been due to early treatment, prior to extensive induction of neuroinflammation. However, the results also point to increased effectiveness of D + Q in maintaining number of microglial processes and length of microglial processes. Furthermore, compared to AV animals, D + Q treatment, but not ABT‐263 treatment, decreased expression of genes linked to positive regulation of glial cell migration and regulation of microglial cell activation. Finally, similar to AV animals, the AA groups continued to exhibit increased expression of genes for TNF signaling, Fc gamma R‐mediated phagocytosis, and glial cell development compared to YNG.

The ability of ABT‐263 to decrease some markers of neuroinflammation may be due to a leaky BBB, permitting increased access of ABT‐263 to the brain. Senescence of vascular cells can result in neurovascular uncoupling and increased susceptibility of the BBB to disruption, which may be prevented by senolytic treatment (Lee et al., 2021; Tarantini et al., 2021; Yamazaki et al., 2016). However, in this case, it would be expected that ABT‐263 should have decreased microglial activation in aged mice (Mehdipour et al., 2021). Furthermore, in the current study, ABT‐263 treatment preserved BBB integrity, reducing expression of TGFβ signaling genes (e.g. *Acvrl1*, *Cldn5*, *Eng*, *Id1*, *Tfgfb1*) associated with BBB disruption (Chen et al., 2020; Senatorov Jr et al., 2019; Figure S2), and preventing the rise in albumin entry into the brain, indicating that it is unlikely that ABT‐263 achieved significant brain levels. Whether hippocampal BBB disruption contributes to cognitive decline during normal aging is debated (Montagne et al., 2015; Verheggen et al., 2020), partly due to differences in techniques employed and variability in BBB leakiness (Banks et al., 2000; Bowman et al., 2018; Goodall et al., 2018; Moinuddin et al., 2000; Senatorov Jr et al., 2019). Importantly, BBB permeability correlates with serum inflammatory markers (Bowman et al., 2018) and increases under conditions that evoke inflammation (Montagne et al., 2022; Varatharaj & Galea, 2017).

Indeed, the reduction in systemic inflammation likely contributed to treatment effects on neuroinflammation and immune response genes in the brain (Balusu et al., 2016; Barter et al., 2020; Norden et al., 2016; Yegla & Foster, 2019). Thus, the increase in TNF signaling genes in the brain of the AA group, which was absent from the ADQ group, may be due to the ability of D + Q to decrease plasma TNFα. In this case, plasma TNFα may underlie increased TNF signaling in the DG of the AA group and TNFα contributes to some markers of inflammation and cell senescence (Budamagunta, Manohar‐Sindhu, et al., 2021).

A divergence in the ability to remove peripheral senescent cells could influence off‐site bystander effects on the brain (Hickson et al., 2019; Xu et al., 2018). However, very few differences were noted in the ability of D + Q and ABT‐263 to decrease senescence in the periphery. Indeed, ABT‐263 may have had a greater effect, decreasing *Cdkn2a* and *Tnfsf11* to a greater extent in the spleen. Previous studies indicate that the ability of D + Q to decrease plasma SASP factors during aging is highly variable (Farr et al., 2017; Hickson et al., 2019; Justice et al., 2019; Krzystyniak et al., 2022; Novais et al., 2021) and little is known about the effect of ABT‐263 on plasma SASP factors. We confirmed an increase in pro‐inflammatory cytokines/chemokines with age and several inflammatory mediators (IL‐1β, IP‐10, and RANTES) decreased following senolytic treatment, with some markers more responsive to D + Q (TNFα) or ABT‐263 (IFNγ, leptin, EGF). Importantly, many of the plasma inflammatory markers decreased by senolytic treatment influence microglia (Hickman et al., 2013), disrupt the BBB, or cross the BBB to disrupt cognition (Banks & Kastin, 1992; Murta et al., 2015; Rahman et al., 2018; Wang et al., 2018; Yan et al., 2022). Moreover, systemic inflammation contributes to brain redox stress that mediates the decline in NMDAR function (Bodhinathan et al., 2010; Kumar et al., 2018; Kumar & Foster, 2013) and increased systemic inflammation during aging is associated with impaired cognitive function (Marksteiner et al., 2011; Scheinert et al., 2015; Serre‐Miranda et al., 2020). Together the results indicate that both treatments were able to remove peripheral senescent cells and decreased systemic inflammation, which likely contributed to preserved BBB integrity, synaptic function, and cognition.

In addition to decreasing basal measures of systemic inflammation, senolytic treatment can inhibit the hyper‐responsive release of SASP factors during acute systemic inflammation (Budamagunta, Manohar‐Sindhu, et al., 2021; Camell et al., 2021). Concern about acute systemic infection on the trajectory of cognitive decline has dramatically increased due to the neurocognitive sequelae associated with acute infections, including COVID‐19 (Choutka et al., 2022; Crivelli et al., 2022; Zhao et al., 2022). The enhanced release of SASP factors can induce senescence in neighboring cells and cytokines/chemokines from the periphery enter the brain, contributing to the trajectory of cognitive decline and brain aging, increasing expression of immune response genes, decreasing synaptic genes, and impairing NMDAR function (Barter et al., 2021; Bettcher & Kramer, 2014; Beydoun et al., 2019; Mavrikaki et al., 2021). Thus, senolytic treatments may preserve cognition by limiting the effects of acute and chronic systemic inflammation.

## 4 CONCLUSION

D + Q and ABT‐263 treatment, over the course of aging, maintained memory to a similar extent. The treatment‐mediated reduction in systemic inflammation likely contributes to maintenance of BBB integrity, decreased immune responses in the brain, and preservation of synaptic connectivity, including maintenance of NMDAR function involved in spatial memory. Differences in brain gene expression for the two treatments are likely due to additional antioxidant effects of quercetin, disparity in brain access of D + Q and ABT‐263, and differential effects on systemic inflammation. Together, the results emphasize the importance of peripheral senescence and systemic inflammation in mediating age‐related brain changes that contribute to cognitive decline.

Senolytic therapy can reverse some late‐life diseases and improve lifespan in mouse models (Musi et al., 2018; Roos et al., 2016; Yousefzadeh et al., 2018). In the current study, treatment was initiated in middle‐age. Thus, the effectiveness of treatment may change with more advanced age, which would presumably have a higher burden of established diseases, increased numbers of senescent cells, and age‐related epigenetic modifications. Finally, sex may determine the effectiveness of treatments that influence aging phenotypes (Casaletto et al., 2020; Strong et al., 2020) and little is known about the effect of senolytic treatment in females and results are mixed (Muralidharan et al., 2022; Novais et al., 2021; Schwab et al., 2022). Thus, future studies should examine effects of senolytic treatment on cognition and brain transcription in females.

## 5 EXPERIMENTAL PROCEDURES

### 5.1 Animals

Procedures and experiments pertaining to animals have been reviewed and approved by the Institutional Animal Care and Use Committee (IACUC) of University of Florida. All the procedures and experiments involving animals were in accordance with the guidelines set forth by the United States Public Health Service Policy on Humane Care and Use of Laboratory Animals. This study utilized male Fischer 344 rats of different ages obtained from the National Institute on Aging through University of Florida animal care services. The animals were maintained in a reverse cycle 12:12 h light/dark schedule. They were provided with ad libitum access to food and water.

### 5.2 Treatments

Rats (total = 108) were allowed to acclimatize to their new animal facility and the reverse light cycle schedule for at least 10 days before the initiation of any procedure. Middle‐aged rats (12 months; total = 86) were divided randomly into three groups of which one group received a vehicle treatment (AV, *n* = 36), another group received a dasatinib (1.2 mg/kg) + quercetin (12 mg/kg) cocktail (ADQ, *n* = 28) while the final group received ABT‐263 (12 mg/kg) treatment (AA, *n* = 22). The dose of D + Q was based on the literature for mice and the dose of ABT‐263 was optimized using a dose/response (0–25 mg/kg, orally) examining cognition and peripheral markers (data not shown). The drugs were dissolved in a vehicle containing 60:30:10 ratio of Phosal 50 PG, PEG400, and ethanol, respectively. Rats were treated via oral gavage for 5 consecutive days with a 2 week break in between two cycles (as depicted in Figure S1). A total of eight cycles of treatment over the span of 6 months were administered before the rats were behaviorally characterized for motor and cognitive performance. A final round of senolytic treatment was administered a week after the completion of the behavioral characterization and rats were euthanized 2 weeks following the final treatment. Young animals (6 months; YNG; *n* = 22) were tested at the same time as older groups.

### 5.3 Behavior

#### 5.3.1 Cue discrimination task

Animals were tested on the cue discrimination and the one‐day version of the spatial watermaze task, starting 1 week following eight cycles of treatment (~6 months after initial treatment). Details of behavior measures have been previously described (Barter et al., 2021; Guidi et al., 2014; Smith et al., 2020). A 1.7 m diameter black circular water tank within a well‐lit room was surrounded by a black curtain. The temperature of the water was maintained at 27 ± 2°C. An escape platform, roughly 1 cm above the water level, held a white visual cue. Noldus EthoVision software was used to record and process data from the trials. Before testing, rats were separated into individual cages. After 20 min of acclimatization to the new cages, the animals were habituated to the pool by letting them swim freely for 30 s. Behavioral training consisted of five training blocks of three trials each and the entirety of cue discrimination training was completed in 1 day. The inter‐trial interval was 20 s and inter‐block interval was 20 min. At the end of each block, the animal was returned to its cage which was placed in front of a heater to prevent hypothermia. Release points, platform, and start locations were randomized for each trial. Rats were given 60 s per trial to find the platform and if they failed to do so, they were gently guided to the platform.

#### 5.3.2 Spatial discrimination task

Three days after the cue discrimination training, animals were trained on the one‐day spatial version of the watermaze to assess their ability to use the distally placed spatial cues to remember and navigate to the location of the submerged platform (Barter et al., 2020; Barter et al., 2021; Foster & Kumar, 2007; Guidi et al., 2014; Guidi, Kumar, & Foster, 2015; Kumar et al., 2018). Bright and contrasting objects were placed on all four sides of the pool to act as distally located spatial cues. The escape platform was submerged 1 cm below the water surface and the platform location was fixed throughout the duration of the spatial discrimination training. The training consisted of five blocks of three trials per block and the start location for each trial was changed randomly for each trial. Each rat was given 60 s to find the location of the platform and if they failed to find the platform within the 60 s, they were gently guided to the platform. The inter‐trial interval was 20 s and the inter‐block interval was 20 min. At the end of each block, the rat was returned to the holding cage which was placed next to a heater to prevent hypothermia.

At the end of the 5th block, an acquisition probe trial was performed. The platform was removed from the pool and each rat was released from the quadrant opposite the goal quadrant, where the platform was initially located. During the probe trial, the rat was allowed to swim freely for 60 s. After the end of the acquisition probe trial, a refresher block of training with the platform placed back into the goal quadrant was administered. 24 h after spatial training, the rat was tested on the retention probe trial where the platform was again removed from the pool and the rat was allowed to swim for 60 s. To quantitatively assess the performance on the probe trials, discrimination index (DI) scores were calculated using the formula [(time spent in goal quadrant − time spent in opposite quadrant)/(time spent in goal quadrant + time spent in opposite quadrant)].

#### 5.3.3 Inhibitory avoidance

To further assess learning and memory, 7 days after the conclusion of the spatial watermaze training, an inhibitory avoidance test was conducted based on the protocols established previously (Foster & Kumar, 2007; Speisman et al., 2013; Zhou et al., 2011). In short, an inhibitory avoidance apparatus (Coulbourn Instruments) comprising two compartments connected by an automatic door was used for this test. One of the chambers was lit by a light while the other chamber was maintained dark. On the training day, one rat at a time was put into the light chamber and was allowed to acclimatize for 90 s. The connecting door was programmed to automatically open at 90 s, allowing the rat to access the dark chamber. The rat was given 10 min to enter the dark chamber and once all four paws of the rat crossed over to the dark chamber, the automatic door was shut and the rat was given a relatively mild electric shock (0.21 mA) for 3 s. This usually elicits a jump or rapid movement response in the rat, which confirms the rat received an electric shock. Five seconds later, the rat was removed from the chamber and returned to its home cage.

On the testing day (24 h after the training trial), the rat was once again placed in the light chamber and allowed to acclimatize for 90 s before the connecting door opened. The rat was then given 10 min to re‐enter the dark chamber at the end of which the rat was returned to their home cage. The latency to re‐enter the dark chamber was recorded and used to assess memory.

#### 5.3.4 Grip strength test

Grip strength was determined as described previously (Carter et al., 2009; Cui et al., 2009; Kumar et al., 2012; Zhou et al., 2011). Briefly, grip strength was assessed using an automated grip strength meter by sensing the peak amount of force an animal applies in grasping the pull bar assembly (Columbus Instruments) The rat was hand‐held by the experimenter using assembly (Columbus Instruments). For each measurement, the rat's forelimbs were gently placed on the bar, the animal grabbed the bar (a reflex response in rodents), and was then drawn along a straight line leading away from the sensor. The rat released the pull bar at some point and the maximum force attained was stored on the digital display. The peak amount of force the animal applied in grasping the pull bar was measured. The mean force (g) was calculated over three trials and was divided by body weight. The mean force (g) was calculated over three trials, separated by 2–4 min.

### 5.4 Hippocampal slice electrophysiological recordings

Two weeks after the eighth cycle of senolytic treatment, rats were sacrificed and electrophysiological recordings were performed on hippocampal slices as described previously (Bodhinathan et al., 2010; Guidi, Kumar, & Foster, 2015; Kumar, 2015; Kumar et al., 2009, 2018, 2019; Kumar & Foster, 2013). The rat was anesthetized using isoflurane before being decapitated using a guillotine. The whole brain was harvested and briefly incubated (~30 s) in a beaker containing pre‐chilled, ice‐cold calcium‐free artificial cerebrospinal fluid (aCSF in mM: NaCl 124, KCl 2, KH_2_PO_4_ 1.25, MgSO_4_ 2, CaCl_2_ 0, NaHCO_3_ 26, and glucose 10). Both hippocampi were then harvested and ~400 μM sections cut parallel to the alvear fibers. These slices were then transferred to the interphase recording chamber where they were incubated in standard aCSF (in mM: NaCl 124, KCl 2, KH_2_PO_4_ 1.25, MgSO_4_ 2, CaCl_2_ 2, NaHCO_3_ 26, and glucose 10), which was continuously oxygenated. The temperature of the aCSF was maintained at 30 ± 0.5°C and a pH of 7.4. fEPSPs from CA3‐CA1 hippocampal synaptic contacts were recorded with glass micropipettes (4–6 MΩ) filled with aCSF. Concentric bipolar stimulating electrodes (outer pole: stainless steel, 200 μm diameter; inner pole: platinum/iridium, 25 μm diameter, Fredrick Haer & Co) were positioned on approximately 1 mm from the recording electrode localized to the middle of stratum radiatum to stimulate CA3 inputs onto CA1. Using an SD9 stimulator (Grass Instruments), field potentials were induced by single diphasic stimulus pulses (100 μs). The signals were amplified, filtered between 1 Hz and 1 kHz, and stored on computer disk for offline analysis (Data Wave Technologies).

Following acquisition of an input/output curve for total synaptic potentials, the NMDAR‐fEPSPs were isolated as previously described (Bodhinathan et al., 2010; Kumar et al., 2019; Kumar & Foster, 2013). Briefly, the hippocampal slices were incubated for ≥60 min in aCSF containing 0.5 mM magnesium (Mg2+), 30 μM 6,7‐dinitroquinoxaline‐2,3‐dione (DNQX) and 10 μM picrotoxin (PTX) to isolate NMDAR‐mediated synaptic response. Following isolation of NMDAR EPSP, input–output curve for NMDAR‐EPSP was generated by applying increasing stimulation intensities.

### 5.5 Tissue harvesting

Animals were deeply anesthetized with isoflurane and decapitated using a guillotine. Brains were then quickly harvested and rinsed with pre‐chilled saline. Using surgical tools, the hippocampi were removed, placed on a dissection tray on ice and the DG sub‐region was further isolated. In addition, peripheral organs, lung, liver, spleen, and kidney were harvested by making a vertical incision on the ventral surface of the carcass. All the tissues were promptly flash‐frozen in liquid nitrogen and stored at −80°C.

### 5.6 Next‐generation RNA sequencing and data analysis

Transcriptional profiles were analyzed in the DG sub‐region of hippocampus from rats belonging to various groups: young (YNG, *n* = 10), aged vehicle (AV, *n* = 12), aged + DQ (ADQ, *n* = 11), aged + ABT‐263 (AA, *n* = 11) treated rats. RNA isolation, library preparation, and transcriptomic sequencing were performed based on the previously published methods (Barter et al., 2019; Ianov et al., 2016, 2017). Briefly, RNA was isolated using RNeasy Lipid Tissue Mini kit (Qiagen, Catalog Number #74804). DNA was eliminated from the samples using an RNase‐Free DNase Kit (Qiagen, Catalog Number #79254). A NanoDrop 2000 spectrophotometer measured RNA concentration and purity and a High Sensitivity RNA ScreenTape in an Agilent 2200 Tapestation system measured RNA integrity number (RIN). RNA with a RIN greater than 8 was spiked with External RNA Controls Consortium (ERCC) control (Thermo Fisher, Catalog Number #4456740), to assess the quality of the library preparation. Dynabeads mRNA DIRECT Micro Kit (Thermo Fisher; Catalog Number #61021) was used for poly (A) selection of mRNA from the isolated total RNA. Using the isolated mRNA, whole transcriptome libraries were prepared with Ion Total RNA‐Seq Kit v2 (Thermo Fisher, Catalog Number #4475936). Ion Xpress barcodes (Thermo Fisher, Catalog Number #4475485) were utilized for multiplex sequencing. The library size distribution and molar concentration were determined using Qubit dsDNA High Sensitivity Assay kit (ThermoFisher, Cat# 32851) and High Sensitivity D1000 Screen tape Kit (5067‐5584) on 2200 TapeStation System (Agilent Technologies, Cat# G2964A) according to the manufacture's protocol. Templates were then prepared on Ion Chef System and sequencing was carried out on an Ion Proton. The RNA‐sequencing data from this study have been uploaded to NCBI's Gene Expression Omnibus under the accession number GSE220971.

For data analysis to obtain the list of DEGs, Partek Flow server was used. FASTQ files were trimmed and aligned to rat (rn6) genome using STAR. The gene counts were normalized using median ratio and any gene with the total number of counts lower than five per sample was excluded from the analysis. DESeq2 was utilized to obtain a list of DEGs. A threshold *p*‐value lower than 0.05 was used as a cut‐off to statistically filter genes. Genes that passed this statistical filter were grouped into “upregulated” and “downregulated” genes which were then separately run through NIH Database for Annotation, Visualization, and Integrated Discovery (DAVID) for gene enrichment and functional annotation clustering analysis. This analysis was limited to cellular components, biological process, and molecular function in the “Direct” and “FAT” categories, and Kyoto Encyclopedia of Genes and Genomes (KEGG). A Benjamini false discovery rate (FDR) of adj‐*p* < 0.05 was used as a threshold to identify significant clusters. Finally, directed analysis (*p* < 0.05) was conducted for some specific comparisons linked to cell senescence.

### 5.7 Immunohistochemistry

The cerebral cortex was fixed in 4% paraformaldehyde for 48 hours after which it was washed with phosphate‐buffered saline and transferred to 70% ethanol solution until processed. The cerebral cortex was then embedded in paraffin and sectioned into 4 μm thick slices, which were then mounted on glass slides. Slides with sections of the cerebral cortex were deparaffinized by incubating three times in xylene for 10 min. These deparaffinized sections were then rehydrated by serially incubating the slides in 100%, 95%, 80%, and 60% ethanol for 5 min in each solution. After rinsing with distilled water, the slides were incubated in citrate buffer at 95°C for 45 min. The slides were then rinsed thrice with 1× tris‐buffered saline with 0.1% Tween (TBST) before incubating in 3% hydrogen peroxide for 10 min. The slides were blocked with 10% goat serum for 1 h and then with rabbit anti‐rat albumin/iba‐1 (diluted 1:250) overnight at 4°C. After being washed three times with 1× TBST for 3 min each, the slides were incubated with goat anti‐rabbit secondary antibody conjugated with HRP for 90 min. The slides were then washed thrice with 1× TBST for 3 min each and were incubated with a solution containing DAB and hydrogen peroxide for 90 s. The slides were then washed with water before incubating with hematoxylin solution for 20 s. The slides were washed again under running water before being dehydrated by serially incubating in 60%, 80%, 95%, and 100% ethanol for 2 min each and xylene for 5 min. The slides were then sealed with mounting media and coverslips. Services from the Molecular Pathology Core at University of Florida were utilized for the timely completion of the immunohistological staining.

The stained sections were blindly scored based on fixed parameters. For albumin staining, 10 random imaged cerebral cortical regions per animal were chosen and the number of albumin‐positive cells per 50 total cells were counted in each region. For iba‐1, multiple parameters such as the size of soma, average length of the longest arm of the processes, and the number of processes per cell were measured using ImageJ. Five random imaged cerebral cortical regions per animal were chosen and five cells per area of cerebral cortex were analyzed.

### 5.8 Plasma cytokine and chemokine analysis

At the time of tissue collection, trunk blood was collected in ethylenediaminetetraacetic acid (EDTA) tubes (*n* = 6 rats per group). Plasma was harvested from the blood by centrifuging the tubes at 1600 *g* for 10 min at room temperature. The plasma was then flash frozen in liquid nitrogen and stored at −80°C for downstream analysis. These plasma aliquots were then subjected to analysis using Rat Cytokine/Chemokine 27‐Plex Discovery Assay by the Eve Technologies Corporation. Cytokine concentrations falling below the threshold of detection were not included. For statistical analysis, the number of samples per group was 3–6.

### 5.9 Quantitative polymerase chain reaction (qPCR)

RNA was isolated from 30 mg of tissue (lung, liver, spleen, and kidney) using RNeasy mini kit (Cat. No. 74106, Qiagen) and was converted into cDNA using a high‐capacity cDNA reverse transcription kit (Cat. No. 4368813, Applied Biosystems) following manufacturer's instructions. Gene expression was then quantified using gene‐specific primers (Table S1) and fast SYBR green master‐mix (Cat. No. 4385617, Applied Biosystems) as per the manufacturer's instructions. GAPDH was used to normalize the expression levels across all samples and the gene expression level in untreated tissues was used as baseline to compare the fold change in expression between groups. Fold change in gene expression was determined using ΔΔCT method.

### 5.10 Statistical analyses

Statistical analyses for measures other than next‐generation sequencing (e.g. behavior, electrophysiology, and plasma cytokines) were performed using Statview software (SAS Institute). Parametric variables were presented as mean ± SD and non‐parametric variables were presented with geometric mean. ANOVAs were employed to examine main effects and interactions. Significant differences were localized using Fischer's PLSD post hoc comparisons (*p* < 0.05). One‐tailed one‐group *t* tests (*p* < 0.05) were performed to determine if the DI scores were above that expected by chance (i.e. DI score = 0). Kruskal–Wallis test was employed for inhibitory avoidance (*p* < 0.05), with Mann–Whitney *U* tests to localize differences (*p* < 0.05).

## AUTHORS CONTRIBUTIONS

VB, designed and performed experiments, analyzed data, constructed illustrations, and wrote the manuscript, AK, designed and performed experiments, analyze data, constructed illustrations, wrote manuscript, AR, performed experiments, analyzed data, and manuscript editing, LB, performed experiments, SMS, performed experiments, YY, performed experiments, DZ, designed experiments, TCF, designed the experiments, analyzed data, wrote the manuscript, and constructed illustrations.

## CONFLICT OF INTEREST STATEMENT

Daohong Zhou is an inventor of two pending patent applications for use of Bcl‐xL PROTACs, synthesized using ABT‐263, as senolytic and antitumor agents. Daohong Zhou is a co‐founder of and has equity in Dialectic Therapeutics, which develops Bcl‐xL PROTACs to treat cancer.

## Supporting information


**Figure S1.** (a) Time course of treatment. Starting at 12 months of age, rats were treated via oral gavage for 5 consecutive days with a 2‐week break in between two cycles. A total of 8 cycles of treatment over the span of 6 months were administered before the rats were behaviorally characterized for motor and cognitive performance. A final round of senolytic treatment was administered a week after the completion of the behavioral characterization and rats were euthanized 2 weeks following the final treatment. (b) Body weight over the course of the study. (c) Grip strength normalized to weight. # = difference from YNG, * = difference from AV, Ϯ = difference from ADQ.

**Figure S2.** Expression levels of genes involved in TGF‐β signaling as obtained from mRNA sequencing (a–e). Normalized gene counts of *tgfb1* (a), *cldn5* (b), *Eng* (c), *acvrl1* (d) and *id1* (e).

**Table S1.** List of primers used for qPCR.

## Data Availability

All data related with these studies is provided as a Supporting Information, and if any other details are needed will be provided on request. GEO accession number: GSE220971.
