# Supplementary figures and images for: Effect of peripheral cellular senescence on brain aging and cognitive decline
Source: Aging Cell. 2023 Mar 23;22(5):e13817. doi: 10.1111/acel.13817 (PMC10186609; doi:10.1111/acel.13817)

Supplemental Figure 1

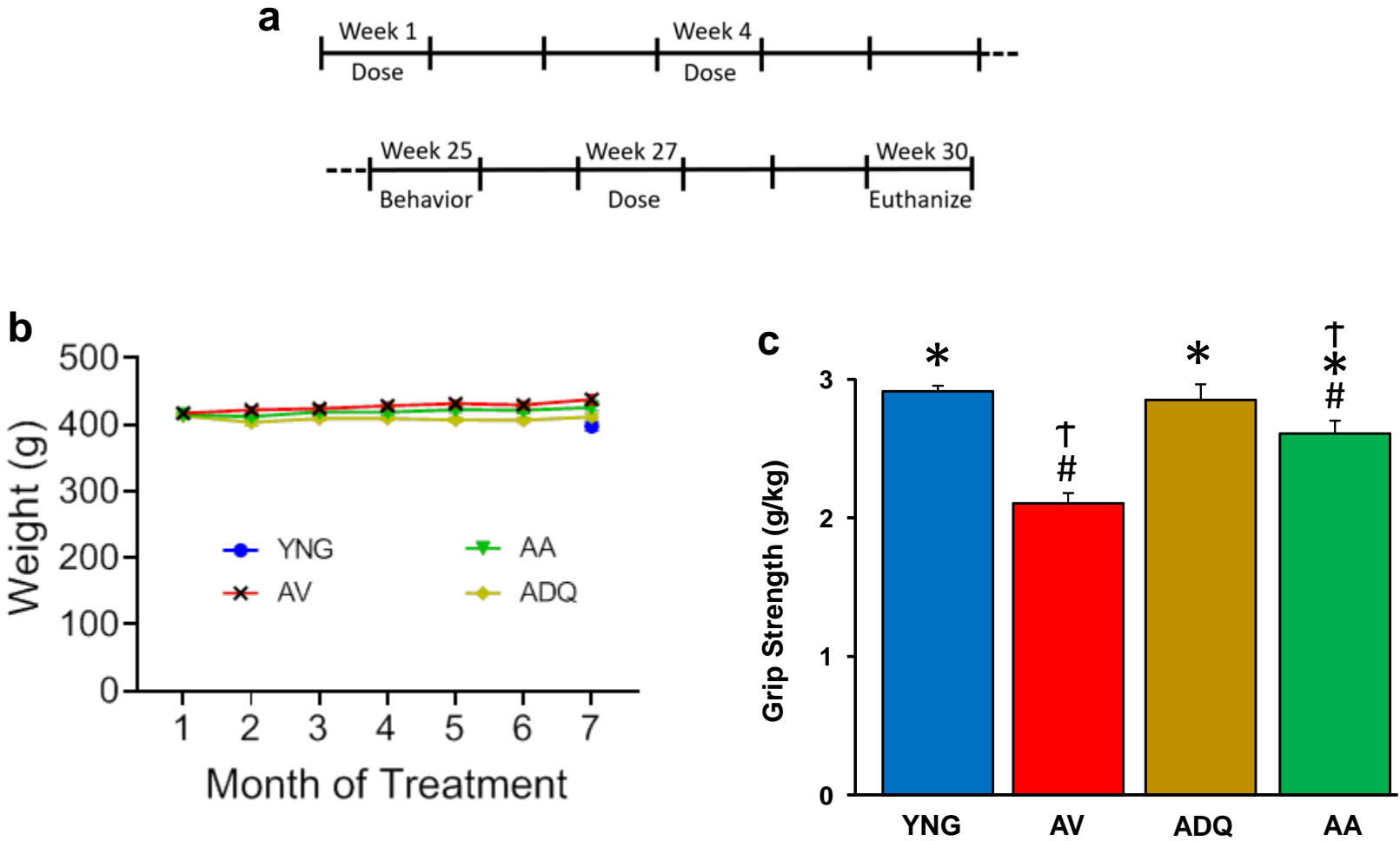

Supplement: Supplementary file 1 — Figure S1. (a) Time course of treatment. Starting at 12 months of age, rats were treated via oral gavage for 5 consecutive days with a 2‐week break in between two cycles. A total of 8 cycles of treatment over the span of 6 months were administered before the rats were behaviorally characterized for motor and cognitive performance. A final round of senolytic treatment was administered a week after the completion of the behavioral characterization and rats were euthanized 2 weeks following the final treatment. (b) Body weight over the course of the study. (c) Grip strength normalized to weight. # = difference from YNG, * = difference from AV, Ϯ = difference from ADQ. [file ACEL-22-e13817-s002.pdf]
