# Supplementary material for: Effect of peripheral cellular senescence on brain aging and cognitive decline
Source: Aging Cell. 2023 Mar 23;22(5):e13817. doi: 10.1111/acel.13817 (PMC10186609; doi:10.1111/acel.13817)
Supplement: Supplementary file 2 — Figure S2. Expression levels of genes involved in TGF‐β signaling as obtained from mRNA sequencing (a–e). Normalized gene counts of tgfb1 (a), cldn5 (b), Eng (c), acvrl1 (d) and id1 (e). [file ACEL-22-e13817-s003.pdf]

Supplemental Figure 2

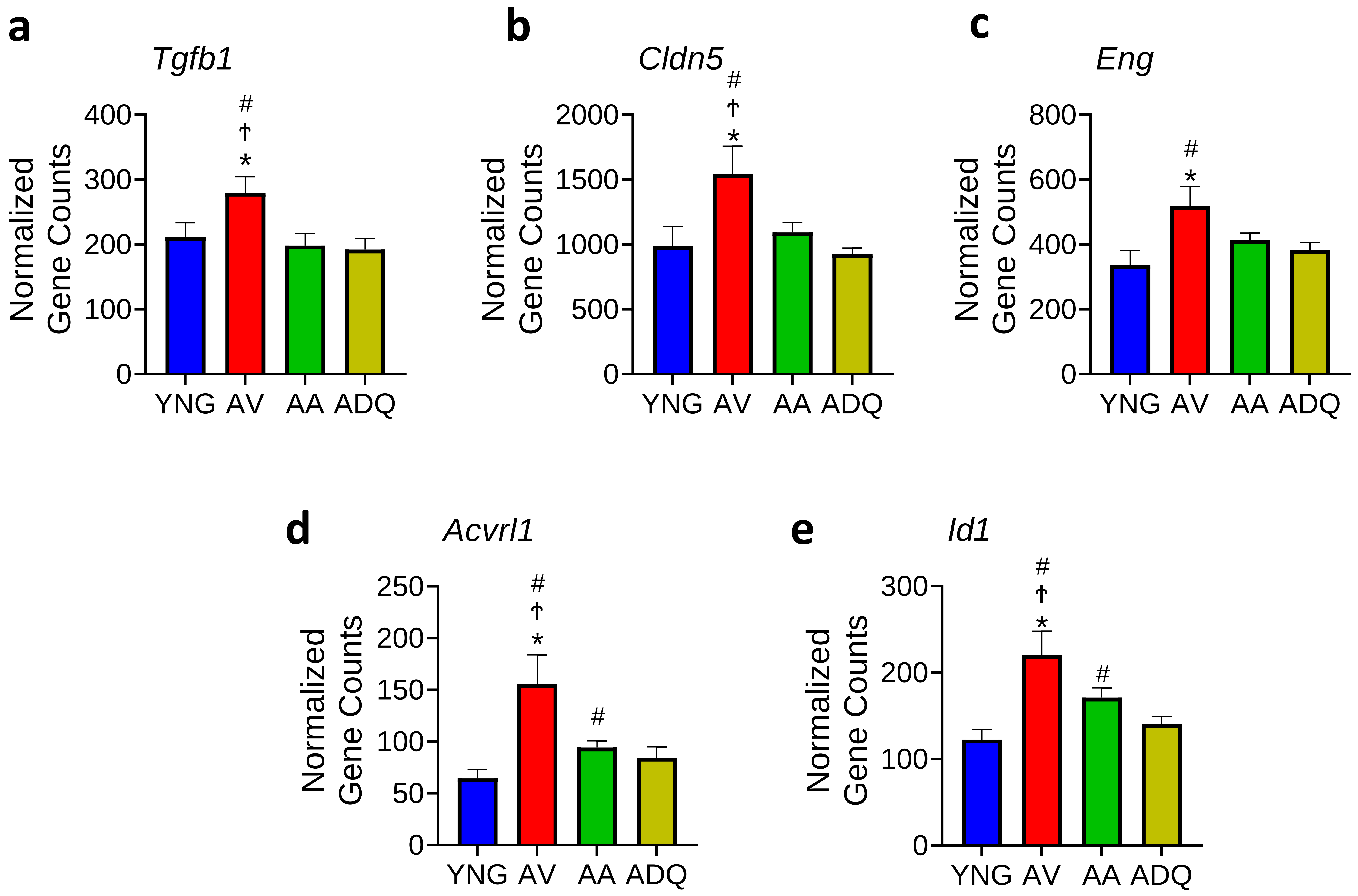

Expression levels of genes involved in TGF-β signaling as obtained from mRNA sequencing (a-e). Normalized gene counts of *tgfb1* (a), *cldn5* (b), *Eng* (c), *acvrl1* (d) and *id1* (e).

# significantly different when compared to YNG
† significantly different when compared to AA
* Significantly different when compared to ADQ
