# Supplementary material for: Effect of peripheral cellular senescence on brain aging and cognitive decline
Source: Aging Cell. 2023 Mar 23;22(5):e13817. doi: 10.1111/acel.13817 (PMC10186609; doi:10.1111/acel.13817)
Supplement: Supplementary file 3 — Table S1. List of primers used for qPCR. [file ACEL-22-e13817-s001.docx]

**Supplementary Table 1. List of primers used for qPCR**.

| **Gene** | **Forward Primer** | **Reverse Primer** |
| --- | --- | --- |
| GAPDH | TCACCACCATGGAGAAGGC | GCTAAGCAGTTGGTGGTGCA |
| IL6 | TAGTCCTTCCTACCCCAACTTCC | TTGGTCCTTAGCCACTCCTTC |
| TNFSF11 | ATGCAGGAGAATGAAACAAGCCT | CTGTGGCCCCACAATGTGTT |
| MMP3 | TGGGAAGCCAGTGGAAATG | CCATGCAATGGGTAGGATGAG |
| CDKN2A | TGCAGATAGACTAGCCAGGGCA | CTTCCAGCAGTGCCCGCA |
| CDKN1A | AGAGCCACAGGCACCATGTC | ACAGACGACGGCATACTTTGC |
